# Selective insulin resistance with differential expressions of IRS-1 and IRS-2 in human NAFLD livers

**DOI:** 10.1038/s41366-018-0062-9

**Published:** 2018-05-01

**Authors:** Midori Honma, Shojiro Sawada, Yoshiyuki Ueno, Keigo Murakami, Tetsuya Yamada, Junhong Gao, Shinjiro Kodama, Tomohito Izumi, Kei Takahashi, Sohei Tsukita, Kenji Uno, Junta Imai, Eiji Kakazu, Yasuteru Kondo, Kei Mizuno, Naoki Kawagishi, Tooru Shimosegawa, Hideki Katagiri

**Affiliations:** 1 Department of Metabolism and Diabetes, Tohoku University Graduate School of Medicine, Sendai, Japan; 2 Department of Gastroenterology, Yamagata University Faculty of Medicine, Yamagata, Japan; 3 Japan Agency for Medical Research and Development, CREST, Tokyo, Japan; 4 Department of Pathology, Tohoku University Graduate School of Medicine, Sendai, Japan; 5 Center for Metabolic Diseases, Tohoku University Graduate School of Medicine, Sendai, Japan; 6 Division of Gastroenterology, Tohoku University Graduate School of Medicine, Sendai, Japan; 7 Division of Transplantation, Reconstruction and Endoscopic Surgery, Tohoku University Hospital, Sendai, Japan

## Abstract

**Background/objective:**

Insulin signals, via the regulation of key enzyme expression, both suppress gluconeogenesis and enhance lipid synthesis in the liver. Animal studies have revealed insulin signaling favoring gluconeogenesis suppression to be selectively impaired in steatotic livers. However, whether, and if so how, such selective insulin resistance occurs in human steatotic livers remains unknown. Our aim was to investigate selective insulin resistance in human livers with non-alcoholic fatty liver disease (NAFLD).

**Subjects/methods:**

We examined mRNA expressions of key molecules for insulin signaling, gluconeogenesis and lipogenesis in human liver biopsy samples obtained from 51 non-diabetic subjects: 9 healthy controls and 42 NAFLD patients, and analyzed associations of these molecules with each other and with detailed pathological and clinical biochemistry data.

**Results:**

In NAFLD patients, insulin receptor substrate (IRS)-2 expression was decreased, while those of key enzymes for gluconeogenesis were increased. These alterations of IRS-2 and gluconeogenesis enzymes were induced both in simple steatosis (SS) and non-alcoholic steatohepatitis (NASH), while these expression levels did not differ between SS and NASH. Furthermore, alterations in the expressions of IRS-2 and gluconeogenesis enzymes showed strong negative correlations and were concurrently induced in the early histological stage of NAFLD. In contrast, fatty acid synthase (FAS) expression was not decreased in NAFLD, despite IRS-2 downregulation, but correlated strongly with IRS-1 expression. Furthermore, no histological scores were associated with these molecules. Thus, IRS-1 signaling, which is not impaired in NAFLD, appears to modulate FAS expression.

**Conclusion:**

These analyses revealed that selective insulin resistance is present in human NAFLD livers and occurs in its early phases. The effect of insulin, during the IRS step, on gene expressions for lipogenesis and gluconeogenesis are apparently distinct and preferential downregulation of IRS-2 may contribute to selective resistance to the suppressive effects of insulin on gluconeogenesis.

## Introduction

Non-alcoholic fatty liver disease (NAFLD) is characterized by hepatic triglyceride accumulation not due to alcohol consumption (<20 g ethanol per day), resulting in steatosis and hepatic inflammation [1]. Approximately 20% of patients with non-alcoholic simple steatosis (SS) will progress to non-alcoholic steatohepatitis (NASH) with histological changes such as lobular inflammation and/or hepatocellular ballooning. Furthermore, ~20% of patients with NASH will progress to cirrhosis [2]. It is well known that nearly all patients with NAFLD have hepatic insulin resistance, which increases the risk of developing type 2 diabetes [3].

The liver is the central organ for whole-body carbohydrate and lipid homeostasis, and hepatic insulin signaling is essential to maintain the metabolism of these nutrients. Insulin secreted in the fed state promotes glycogen synthesis and lipogenesis but suppresses hepatic glucose production by inhibiting gluconeogenesis and glycogenolysis [4]. On the other hand, suppression of insulin secretion in fasted states promotes gluconeogenesis and glycogenolysis, resulting in normoglycemia. Insulin binds to the insulin receptor (IR) and activates its tyrosine kinase, thereby promoting tyrosine kinase phosphorylation of IR substrates (IRSs), such as IRS-1 and IRS-2, both of which are abundantly expressed in hepatocytes. Tyrosine-phosphorylated IRS-1 and IRS-2 bind to and activate phosphatidylinositol-3-kinase, subsequently activating a serine/threonine kinase Akt [5]. Akt-dependent phosphorylation and nuclear exclusion of forkhead box-containing protein O subfamily-1 suppress expressions of glucose-6-phosphatase (G6Pase) and phosphoenolpyruvate carboxykinase (PEPCK), resulting in the inhibition of hepatic glucose output [6]. Moreover, insulin stimulates the pathway through the upregulation of fatty acid synthase (FAS) [7] as well as upregulating glucokinase (GCK), which catalyzes the first and rate-limiting step of glycolysis, thereby increasing hepatic lipogenesis [8] (Supplementary Figure 1).

Major impacts on insulin signaling are observed under conditions of hepatic steatosis. The molecular mechanism of the alteration in insulin signaling in the liver has been intensively investigated using a number of experimental animal models of obesity and/or hepatic steatosis. First, hepatic IRS-2 expression was found to be downregulated in these animal models, contributing to the development of hepatic insulin resistance. In addition, insulin-induced downregulations of G6Pase and PEPCK are known to be impaired, resulting in enhanced gluconeogenesis [6]. In contrast to impairment of the suppressive effects of insulin on gluconeogenesis, insulin signaling through the hepatic lipogenic pathway is reportedly not suppressed, instead being stimulated [7, 9]. Thus, insulin resistance in the steatotic liver is considered to be limited to the pathway involving suppression of hepatic glucose production, which is referred to as selective insulin resistance [10]. However, the proposed molecular mechanism of selective insulin resistance is based on experimental data obtained from animal models, mainly mice fed a high-fat diet [11]. In contrast, human data on hepatic gene expressions, including IRS-2 [12–14] and gluconeogenic enzymes [15, 16], in patients with NAFLD are scarce and controversial.

Therefore, in the present study, we aimed to investigate hepatic gene expressions involved in insulin signaling integrity for comparison between healthy subjects and NAFLD patients. We further examined the relationships among expressions of these key molecules, the histological severity of NAFLD and clinical blood biochemistry data. On the basis of the results obtained, we herein propose a molecular mechanism underlying selective insulin resistance.

## Materials and methods

### Subjects

A retrospective analysis of the clinical data and histological information was performed on patients with biopsy-proven NAFLD during the period from September 2005 to April 2014 in Tohoku University Hospital and Yamagata University Hospital. Patients were excluded from the analysis if they consumed more than 20 g of alcohol per day, or had evidence of liver diseases with other etiologies, such as viral hepatitis, autoimmune hepatitis, primary biliary cirrhosis, hemochromatosis, drug-induced liver disease and other secondary hepatic diseases. In total, 82 patients were included in the analysis. Because our aim was to investigate selective insulin resistance in NAFLD, 38 patients who had been diagnosed as having diabetes were excluded. Diabetes was defined as HbA_1c_ ≥ 6.5% (48 mmol/mol), fasting plasma glucose ≥ 126 mg/dL or random plasma glucose ≥ 200 mg/dL (11.1 mmol/L), or currently receiving medical treatment for diabetes, according to the American Diabetes Association criteria for the diagnosis of diabetes [17]. Two patients who had been taking eicosapentaenoic acid (EPA) were also excluded from the analysis, because EPA reportedly exerts beneficial effects on hepatic steatosis [18] and steatohepatitis [19] by reducing the expression of sterol responsive element binding protein-1c (SREBP-1c) [20]. Thus, ultimately, we analyzed 42 NAFLD patients. Diagnoses of NAFLD were histologically confirmed in all of these patients, as described below. As controls, we retrospectively enrolled 9 healthy subjects who had been donors for liver transplantation during the same period in Tohoku University Hospital. All control subjects were confirmed to have normal liver functions and histologically normal findings. The Ethics Committee at the Tohoku University School of Medicine approved the research protocol, which was performed in accordance with World Medical Association Declaration of Helsinki. Patients gave written informed consent prior to inclusion in the study.

### Histological liver analyses

Routine needle liver biopsies were performed in all patients and wedge liver biopsies were performed in all donors for histological analyses. Formalin-fixed, paraffin-embedded (FFPE) liver sections were stained with hematoxylin-eosin and Masson’s trichrome. A single highly experienced pathologist (K.M.), unaware of the gene expression data, evaluated all of the biopsy specimens, confirmed the diagnoses and classified NAFLD patients according to the classification by Matteoni et al [21], as follows: type 1: fatty liver alone, type 2: fat accumulation and lobular inflammation, type 3: fat accumulation and ballooning degeneration, or type 4: fat accumulation, ballooning degeneration and either Mallory-Denk bodies or fibrosis. Type 1 and 2 were classified as SS, types 3 and 4 as NASH. As a result, this study included 19 patients with SS and 23 with NASH. In addition, histological features were graded semi-quantitatively according to Kleiner’s score [22], which consists of the degree of steatosis (0–3 points), lobular inflammation (0–3 points) and hepatocellular ballooning (0–2 points) as well as Brunt’s staging classification [23], which represents the degree of fibrosis (0–4 points). Additionally, we calculated the NAFLD activity score as the sum of points for steatosis, lobular inflammation and hepatocellular ballooning [22]. The patients were classified into four groups: no steatosis (0 points), simple steatosis (1–2 points), borderline-NASH (3–4 points) and NASH (5–8 points).

### Biochemical analyses

Fasting plasma glucose, serum insulin, total cholesterol, triglycerides, aspartate aminotransferase (AST) and alanine aminotransferase (ALT), type IV collagen, hyaluronic acid and HbA_1c_ were measured in patients with NAFLD. Fasting plasma glucose, serum total cholesterol, triglycerides, AST, ALT, and HbA_1c_ were measured in healthy control subjects. Some of the laboratory data were lacking in some of the patients and controls. HOMA-IR was calculated as fasting glucose (mg/dL) x fasting insulin (µU/mL)/405. Since the HOMA-IR cut-off point for identifying insulin resistance in healthy Japanese was reported to be 1.7 [24], subjects with HOMA > 1.7 were considered to have insulin resistance in the present study.

### Gene expression analyses

Total RNA was isolated from samples of FFPE liver tissues (10 µm sections) by using an RecoverAll^TM^ Total Nucleic Acid Isolation Kit for FFPE (Applied Biosystems, Foster City, CA) according to the manufacture’s instructions. FFPE samples were deparaffinized using a series of xylene and ethanol washes, then subjected to a rigorous protease digestion for recovery of RNA. The recovered RNAs were suitable for real-time RT-PCR analysis, based on past reports [25, 26]. cDNA was synthesized with approximately 300 ng of total RNA using a High Capacity cDNA Reverse Transcription Kit (Applied Biosystems, Foster City, CA). With 60% amounts of the obtained cDNA, we performed cDNA pre-amplification reactions with primers of target genes using the Taqman Preamplification Master Mix Kit (Applied Biosystems Foster City, CA) in order to increase the sensitivity of real-time PCR analysis of the FFPE samples. The amplified products were 1/20 diluted, and quantitative PCR was performed using an ABI 7700 sequence detection system and Taqman PCR Master Mix (Applied Biosystems Foster City, CA). The following ABI-inventoried TaqMan gene expression assays were used; IR, IRS-1, IRS-2, PEPCK, G6Pase, GCK and FAS. In addition, expressions of two isoforms of IR, IR type A (IR-A) and IR type B (IR-B), were compared using isoform specific ABI-inventoried TaqMan gene expression assays. Cyclin-dependent kinase inhibitor 1B (CDKN1B) was used as an internal control in accordance with the manufacturer’s recommendation. Relative changes in gene expressions were determined using the 2^-ΔΔCt^ method. ΔCt refers to the cycle number at which the transcripts were detectable (Ct) normalized to the cycle number of CDKN1B [27]

### Statistical methods

The presence or absence of a normal distribution was verified using the Shapiro-Wilk W test. The normalized mRNA levels were log-transformed before data analysis due to non-normal distributions. Data, expressed as means ± SEM, were compared by ANOVA with Tukey-Kramer post hoc testing, and correlations were assessed employing Pearson’s test. A level of *P* < 0.05 was considered significant. Analyses were carried out with JMP 11.2 Pro statistical analysis software (SAS Institute, Cary, NC). HbA_1c_ values were missing for 5 subjects (3 control and 2 SS subjects). Fasting serum insulin and HOMA-IR values were missing for 3 of the SS subjects, serum type IV collagen values were missing for 17 subjects (11 SS and in 5 NASH subjects), and serum hyaluronic acid values were missing for 15 subjects (12 SS and 2 NASH subjects). The sample size calculation was based on a previous study comparing hepatic IRS-2 mRNA expression between NAFLD and normal subjects [12]. Assuming an 80% difference with a standard deviation of 45% between NAFLD and subjects free of liver disease, a total of 13 subjects would be required at a 5% significance level and 80% power. As some clinical values were missing due to the data having been retrospectively collected, sample size calculations were readjusted. Sample size calculations were performed using JMP 11.2 Pro statistical analysis software (SAS Institute, Cary, NC).

## Results

### Clinical characteristics

Table 1 shows the clinical characteristics of 9 control subjects, 19 SS subjects, and 23 NASH subjects. Body mass index values were significantly higher in patients with SS and NASH than in the control group. According to the guidelines proposed by the International Obesity Task Force of the World Health Organization (IOTF-WHO), individuals with BMI ≥ 25 in Asian populations are obese [28]. Based on these guidelines, the prevalences of obesity were higher in the SS and NASH groups than in the no steatosis group. The prevalence of hypertension, defined as systolic blood pressure ≥ 140 mmHg and/or diastolic blood pressure ≥ 90 mmHg [29], or taking antihypertensive drugs, also rose with progression of NAFLD (Table 1). AST and ALT levels in the SS and NASH groups were significantly higher than those in the control group. Serum type IV collagen and hyaluronic acid levels in patients with NASH were above the upper limit of the normal range [ < 140 ng/mL and < 50 ng/mL, respectively]. These biochemical data support the pathological classification of NAFLD. HbA_1c_ levels in the NASH group were significantly higher than those in the control group, although these levels did not exceed the upper limit of the normal range. Fasting serum insulin levels in the NASH group were significantly higher than those in the SS group, while fasting plasma glucose levels were similar among the control, SS and NASH groups. Notably, the HOMA-IR of patients with SS and NASH were 3.5 ± 0.7 and, 5.3 ± 0.6, respectively, both above the upper limit of the normal range. These findings suggest the development of systemic insulin resistance in NAFLD patients.

**Table 1:** Anthropometric and biochemical data

	Control	SS	NASH
Male / female (*n*)	6 / 3	13 / 6	8 / 15
Age (years)	36.4 ± 4.3	47.5 ± 3.0	55.3 ± 3.0^a^
Body mass index (kg/m^2^)	22.0 ± 1.3	27.3 ± 0.9^a^	27.3 ± 0.8^a^
Prevalence of obesity (%)	11.1	68.4	78.3
Prevalence of hypertension (%)^b^	0.0	25.0	39.1
HbA_1c_ (%)^c^	5.3 ± 0.1	5.7 ± 0.1	5.8 ± 0.1^d^
HbA_1c_ (mmol/mol)^c^	34.4 ± 1.3	38.6 ± 1.0	40.1 ± 1.0^d^
Fasting plasma glucose (mg/dL)	93.0 ± 3.4	96.9 ± 2.3	98.6 ± 2.1
Fasting serum insulin (µU/mL)^e^	**—**	13.9 ± 2.8	21.9 ± 2.3^f^
HOMA-IR^e^	**—**	3.5 ± 0.7	5.3 ± 0.6^f^
AST (IU/L)	17.6 ± 8.1	36.7 ± 5.6^a^	67.3 ± 4.3^a^
ALT (IU/L)	18.0 ± 13.0	67.8 ± 9.0^a^	74.8 ± 6.9^a^
Total cholesterol (mg/dL)	187.8 ± 10.0	210.4 ± 9.9	185.3 ± 9.0
Triglycerides (mg/dL)	104.2 ± 24.1	158.1 ± 16.6	144.5 ± 15.2
Serum type IV collagen (ng/mL)^g^	**—**	94.6 ± 17.7	162.3 ± 11.7^h^
Serum hyaluronic acid (ng/mL)^i^	**—**	48.0 ± 43.5	105.6 ± 25.5

Data are means ± SEM or numbers (*n*)

*P* was determined by ANOVA with Tukey–Kramer post hoc testing

*HOMA-IR* homeostatic model assessment insulin resistance, *AST* aspartate aminotransferase, *ALT* alanine aminotransferase, *SS* simple steatosis, *NASH* non-alcoholic steatohepatitis

^a^*P* < 0.01 vs. control

^b^Available for all 9 control subjects, 12 of the 19 SS subjects and 22 of 23 NASH subjects

^c^Available for 6 of the 9 control subjects, 17 of the 19 SS subjects and all 23 NASH subjects

^d^*P* < 0.05 vs. Control

^e^Available for 16 of the 19 SS subjects and all 23 NASH subjects

^f^*P* < 0.05 vs. SS

^g^Available for 8 of the 19 SS subjects and 18 of the 23 NASH subjects

^h^*P* < 0.01 vs. SS

^i^Available for 7 of the 19 SS subjects and 21 of the 23 NASH subjects

### Histological liver analyses

The histological features of the liver biopsy samples were semi-quantitatively scored according to the Kleiner’s scoring system [22] and Brunt’s staging classification [23] (Table 2). Kleiner’s score consists of the degree of steatosis, lobular inflammation and hepatocellular ballooning and Brunt’s staging classification [23] represents the degree of fibrosis. The control group showed virtually no features of steatosis, lobular inflammation, ballooning or fibrosis. The degrees of lobular inflammation, ballooning and fibrosis were markedly higher in the NASH than in the SS group, while the degrees of steatosis were similar in these two groups.

**Table 2:** Histological characteristics

Histological feature	Score	Control (*n* = 9)	SS (*n* = 19)	NASH (*n* = 23)
		Number of biopsies (%)		
Steatosis	0 = None (<5%)	9 (100%)	0 (0.0%)	0 (0.0%)
	1 = Mild (5–33%)	0 (0.0%)	11 (57.9%)	14 (60.9%)
	2 = Moderate (>33–66%)	0 (0.0%)	6 (31.6%)	9 (39.1%)
	3 = Severe (>66%)	0 (0.0%)	2 (10.5%)	0 (0.0%)
Lobular inflammation	0 = No foci	9 (100%)	1 (5.3%)	0 (0.0%)
	1 = <2 foci	0 (0.0%)	15 (78.9%)	11 (47.8%)
	2 = 2–4 foci	0 (0.0%)	3 (15.8%)	11 (47.8%)
	3 = >4 foci	0 (0.0%)	0 (0.0%)	1 (4.0%)
Ballooning	0 = None	9 (100%)	15 (78.9%)	1 (4.3%)
	1 = Few balloon cells	0 (0.0%)	4 (21.1%)	17 (73.9%)
	2 = Prominent ballooning	0 (0.0%)	0 (0.0%)	5 (21.7%)
Fibrosis	0 = No	9 (100%)	5 (26.3%)	0 (0.0%)
	1 = Mild, perisinusoidal	0 (0.0%)	11 (57.9%)	2 (8.7%)
	2 = Perisinusoidal and portal/periportal	0 (0.0%)	3 (15.8%)	6 (26.1%)
	3 = Bridging fibrosis	0 (0.0%)	0 (0.0%)	10 (43.5%)
	4 = Cirrhosis	0 (0.0%)	0 (0.0%)	5 (21.7%)

The distributions of the patients according to the degrees of steatosis, lobular inflammation, ballooning and fibrosis are presented as absolute numbers (*n*) and percentages (%) for all 51 subjects in this study

*SS* simple steatosis, *NASH* non-alcoholic steatohepatitis

### Selective resistance to the effects of insulin on hepatic gluconeogenesis suppression, but not lipogenesis promotion, was demonstrated in human NAFLD

**Fig. 1:**
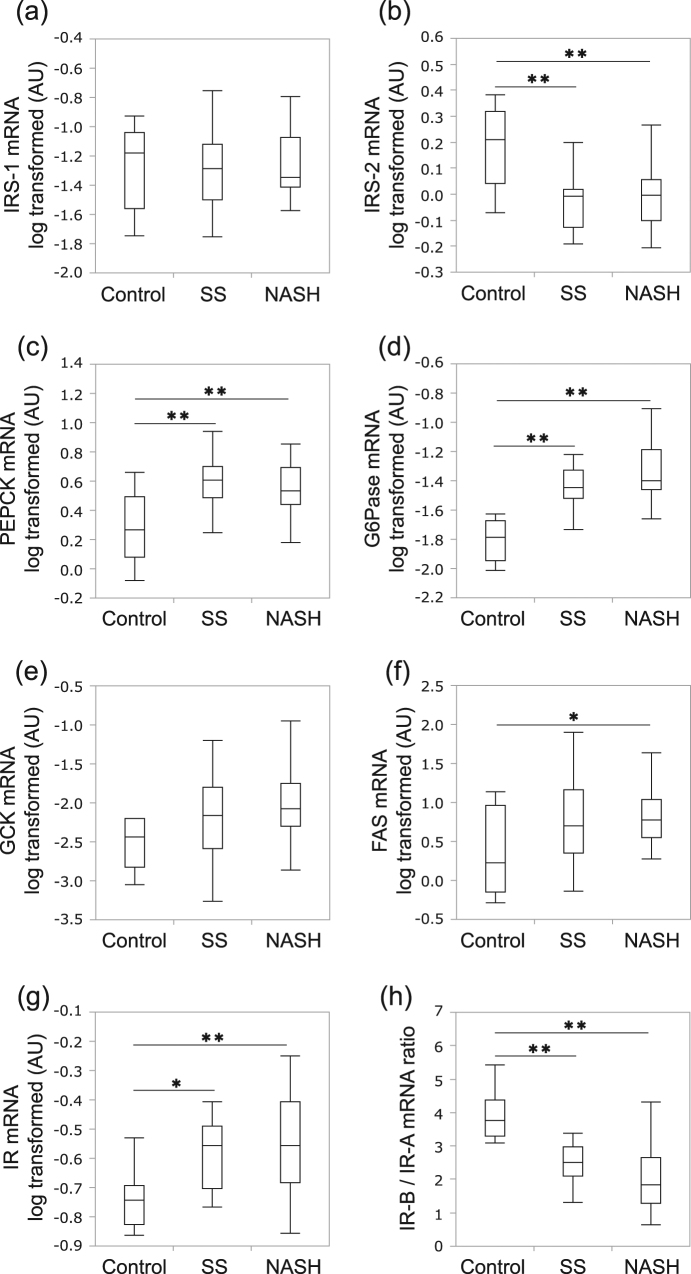
Gene expression levels in the livers of the three groups. The hepatic gene expression levels were compared among the control, SS, and NASH groups. Individual genes are normalized to CDKN1B mRNA levels. IRS-1 (**a**), IRS-2 (**b**), PEPCK (**c**), G6Pase (**d**), GCK (**e**), FAS (**f**), IR (**g**), and IR-B/IR-A ratio (**h**). The data from 51 human liver biopsies are shown. The top and bottom of each box indicate the 1st and 3rd quartiles (interquartile range, IGR), and the band inside the box is the median. The ends of the whiskers represent the minima and maxima without outliers. The value of *P* was determined by ANOVA with Tukey–Kramer post hoc testing. (*P < 0.05, **P < 0.01)

To examine the effects of insulin on gluconeogenesis suppression as well as lipogenesis promotion in the livers of NAFLD patients, we selected key insulin signaling molecules, IRS-1 and IRS-2, and key enzymes for gluconeogenesis, PEPCK and G6Pase, for glycolysis, GCK, and for lipogenesis, FAS. The levels of mRNA expressions of IRS-1 [30], IRS-2 [30], PEPCK [31] and G6Pase [32] reportedly reflect their functions. In addition, hepatic GCK [33] and FAS [34] activities are regulated on the transcriptional level under steatotic conditions. Therefore, we analyzed the hepatic gene expressions of these molecules as well as those of IR and its isoforms (IR-A and IR-B) in 9 healthy control subjects, 19 SS and 23 NASH patients. Although hepatic IRS-1 mRNA levels did not differ among the three groups (Fig. 1a), hepatic IRS-2 mRNA levels were significantly lower in the SS and NASH groups than in the controls (Fig. 1b). Hepatic PEPCK and G6Pase mRNA expressions were significantly higher (Fig. 1c, d), while GCK mRNA expression tended to be higher, in the SS and NASH groups than in the controls (Fig. 1e). On the other hand, hepatic FAS mRNA levels were significantly higher in the NASH than in the control group (Fig. 1f). These findings using human biopsy samples indicate that IRS-2 is downregulated, while IRS-1 expression is unaltered, under NAFLD conditions. In addition, insulin signaling favoring the downregulations of genes for gluconeogenesis was impaired, while that for lipogenic gene upregulation was not impaired, instead being enhanced. Thus, selective resistance to the suppressive effects of insulin on gluconeogenesis, but not lipogenesis promotion, was demonstrated in human NAFLD.

The expression of IR mRNA was lowest in the controls, followed by the SS group, and finally highest in the NASH group (Fig. 1g). However, IR-B/IR-A ratios were highest in the controls, followed by the SS group, and finally lowest in the NASH group (Fig. 1h). These results suggest that IR-A was selectively up-regulated, with the progression of NAFLD in human subjects.

We further analyzed the hepatic gene expressions in 9 healthy subjects and 42 NAFLD patients, divided into groups according to their NAFLD activity scores [22]. There were 9 subjects with no steatosis (NAFLD activity score of 0), 11 with simple steatosis (1–2 points), 23 with borderline-NASH (3–4 points) and 8 with NASH (5–8 points). Although hepatic IRS-1 mRNA levels did not differ among the four groups (Supplementary Figure 2a), hepatic IRS-2 mRNA levels were significantly lower in subjects with simple steatosis, borderline-NASH and NASH than in those with no steatosis (Supplementary Figure 2b). Hepatic PEPCK and G6Pase mRNA expressions were significantly higher (Supplementary Figure 2c, d), while GCK and FAS mRNA expression tended to be higher, in the simple steatosis, borderline-NASH and NASH groups than in the no steatosis group (Supplementary Figure 2e, f). The expression of IR mRNA was significantly higher in the borderline-NASH and NASH groups than in those free of steatosis (Supplementary Figure 2g). However, IR-B/IR-A ratios were highest in the no steatosis group, followed by the simple steatosis, borderline-NASH, and finally lowest in the NASH group (Supplementary Figure 2h). IRS-2, PEPCK, G6Pase and IR expressions did not differ among the simple steatosis, borderline-NASH and NASH groups. The results were quite similar to those obtained using the classification system developed by Matteoni et al. [21] (Fig. 1). Collectively, these findings suggest that the hepatic change associated with simple steatosis, rather than NAFLD progression causes these alterations in the expressions of insulin signaling molecules and insulin-target enzymes.

### Hepatic IRS-1 and IRS-2 gene expressions were strongly linked to lipogenic and gluconeogenic gene expressions, respectively

**Fig. 2:**
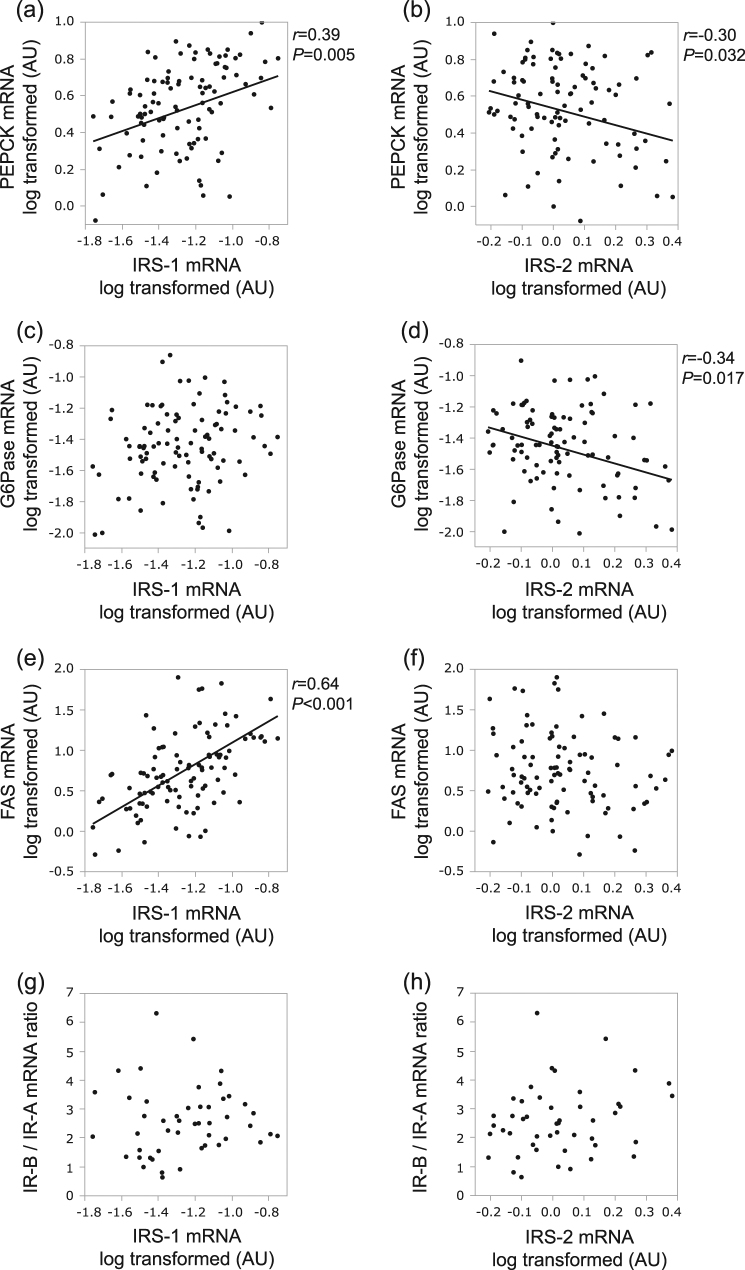
Hepatic IRS-1 and IRS-2 mRNA levels correlated with gluconeogenesis and lipogenesis gene expressions. The scatter plots show the relationship of IRS-1 mRNA levels with PEPCK (**a**) or G6Pase (**c**) or FAS (**e**) mRNA levels or the IR-B/IR-A mRNA ratio (**g**), and the relationship of IRS-2 mRNA levels with PEPCK (**b**) or G6Pase (**d**) or FAS (**f**) mRNA levels or the IR-B/IR-A mRNA ratio (**h**). The data from 51 human liver biopsies are shown. Gene expressions are normalized to CDKN1B mRNA levels. The lines indicate the linear regressions between the parameters. The value of *P* was determined by Pearson’s test

We next examined the relationships between IRS-1 or IRS-2 mRNA levels and gluconeogenesis or lipogenesis genes in all of our study subjects. Interestingly, the hepatic IRS-1 mRNA levels showed a strong positive association with FAS mRNA (*r* = 0.64, *P* < 0.001) (Fig. 2e), but was not negatively associated with either PEPCK or G6Pase mRNA level. For PEPCK, IRS-1 showed a paradoxically positive association (*r* = 0.39, *P* = 0.005) (Fig. 2a). In contrast, hepatic IRS-2 mRNA levels were negatively associated with both PEPCK (*r* = −0.30, *P* = 0.032) (Fig. 2b) and G6Pase (*r* = −0.34, *P* = 0.017) mRNA levels (Fig. 2d). However, no significant association was observed between IRS-2 and FAS expressions. These results suggest that IRS-1 expression is involved in FAS upregulation, while IRS-2 downregulation plays important roles in the impaired suppression of PEPCK and G6Pase. In addition, we examined the relationships between IRS-1 or IRS-2 mRNA levels and the IR-B/IR-A mRNA ratio. However, neither IRS-1 nor IRS-2 showed any significant association with the IR-B/IR-A ratio (Fig. 2g, h). Taken together, these observations indicate that the effects of insulin on lipogenic and gluconeogenic gene expressions are likely to be distinguished from each other at the IRS step and selective downregulation of IRS-2 may contribute to selective insulin resistance, i.e., there is an impairment adversely impacting the capacity of insulin to suppress gluconeogenesis.

### Correlations of hepatic gene expressions with clinical parameters

We analyzed the correlations between hepatic gene expressions and clinical parameters (Table 3). BMI was negatively associated with IRS-2 mRNA (*r* = −0.32, *P* < 0.01), while being positively associated with expressions of PEPCK (*r* = 0.34, *P* < 0.05) and G6Pase (*r* = 0.38, *P* < 0.01). Serum AST levels were positively associated with G6Pase mRNA (*r* = 0.50, *P* < 0.01). Serum ALT levels were negatively associated with IRS-2 mRNA levels (*r* = −0.39, *P* < 0.01), and were positively associated with PEPCK mRNA (*r* = 0.30, *P* < 0.05) and G6Pase mRNA (*r* = 0.49, *P* < 0.01). Serum total cholesterol levels were negatively associated with GCK mRNA levels (*r* = −0.28, *P* < 0.05).

**Table 3:** Correlation coefficients for gene expressions and clinical parameters and histological scores

	IRS-1	IRS-2	PEPCK	G6Pase	GCK	FAS	IR
Clinical parameters							
Body mass index (kg/m^2^)	0.06	−0.32^a^	0.34^b^	0.38^a^	0.13	0.17	−0.01
AST (IU/L)	−0.18	−0.15	0.15	0.50^a^	0.25	0.14	0.26
ALT (IU/L)	−0.09	−0.39^a^	0.30^b^	0.49^a^	0.27	0.11	0.09
Total cholesterol (mg/dL)	−0.04	−0.15	−0.01	−0.13	−0.28^b^	−0.12	−0.18
Triglycerides (mg/dL)	0.12	−0.13	0.26	0.25	0.05	0.08	−0.03
Histological scores							
Steatosis (0–3 points)	−0.17	−0.54^a^	0.38^a^	0.50^a^	0.11	0.04	0.39^a^
Lobular inflammation (0–3 points)	−0.05	−0.35^b^	0.33^b^	0.59^a^	0.33^b^	0.22	0.35^b^
Ballooning (0–2 points)	−0.07	−0.05	0.09	0.43^a^	0.10	0.13	0.24
Fibrosis (0–4 points)	0.08	−0.15	0.30^b^	0.60^a^	0.19	0.19	0.44^a^

Correlations between gene expressions and clinical biochemistry data were assessed employing Pearson’s test

Correlations between gene expressions and the degrees of steatosis, lobular inflammation, ballooning and fibrosis were assessed employing Pearson’s test

The data from 51 human liver biopsies are shown

*AST* aspartate aminotransferase, *ALT* alanine aminotransferase, *IRS-1* insulin receptor substrate-1, *IRS-2* insulin receptor substrate-2, *PEPCK* phosphoenolpyruvate carboxylase, *G6Pase* glucose-6-phosphatase, *GCK* glucokinase, *FAS* fatty acid synthase, *IR* insulin receptor

^a^*P* < 0.01

^b^*P* < 0.05

### Correlations of hepatic gene expressions with the histological severities of NAFLD components

We next analyzed the correlations between hepatic gene expressions and histological severities of NAFLD components in all subjects (Table 3). The severities of steatosis and lobular inflammation correlated negatively with hepatic IRS-2 mRNA levels (*r* = −0.54, *P* < 0.01, and *r* = −0.35, *P* < 0.05, respectively) and positively with PEPCK (*r* = 0.38, *P* < 0.01, and *r* = 0.33, *P* < 0.05, respectively), and G6Pase (*r* = 0.50, *P* < 0.01, and *r* = 0.59, *P* < 0.01, respectively). Thus, resistance to the suppressive effects of insulin on gluconeogenesis apparently begins in the early phases of NAFLD. In contrast, neither IRS-1 nor FAS mRNA levels correlated significantly with the severity of any of the histological features, indicating that insulin signaling for the upregulation of lipogenic enzyme expression is independent of the histological severity of NAFLD. In addition, while ballooning (*r* = 0.43, *P* < 0.01) and fibrosis (*r* = 0.60, *P* < 0.01) correlated positively with G6Pase expression, associations with IRS-2 and PEPCK were weak or absent. Taken together with the finding that ALT, which was already elevated in the SS group (Table 1), is the parameter most strongly associated with IRS-2/PEPCK/G6Pase expressions (Table 3), selective insulin resistance associated with IRS-2 downregulation is likely to occur in the early stages of NAFLD.

## Discussion

One of the important findings of the present study is downregulation of IRS-2 along with upregulation of PEPCK and G6Pase in humans with NAFLD. IRS-2 downregulation was observed in both the SS and the NASH group, but IRS expressions did not differ between the SS and the NASH groups. Similarly, upregulations of PEPCK and G6Pase were observed in both the SS and the NASH group, but the PEPCK and G6Pase expressions did not differ between SS and NASH groups. Thus, IRS-2, PEPCK and G6Pase expressions are concurrently altered in the early stage of NAFLD. In animal studies using obese models such as mice fed a high-fat diet [9, 11, 35], ob/ob mice [7, 36] and Zucker fatty rats [30], levels of IRS-2 mRNA were shown to be decreased in contrast to those of IRS-1. PEPCK and G6Pase are two critical rate-limiting gluconeogenic enzymes, the expressions of which are suppressed by insulin at the transcriptional level [37], but PEPCK and G6Pase mRNA levels in liver are reportedly increased in insulin-resistant animal models [38, 39]. This led to speculation that the decreased IRS-2 in hepatic steatosis is the main contributor to impairment of the insulin signaling that governs the suppression of hepatic glucose production [40]. On the other hand, human data on the hepatic expressions of IRS-2, PEPCK, and G6Pase in NAFLD are scarce and no consensus has yet been obtained. For instance, IRS-2 mRNA expression in liver biopsy samples from NAFLD [9, 12] and NASH [13] subjects were reported to be lower than in those from subjects with normal livers, while there is a report indicating that IRS-2 mRNA expression increased progressively with the severity of NASH [14]. There is a report indicating PEPCK and G6Pase mRNA levels in liver biopsy samples from NASH patients to be higher than in the normal liver [15], while G6Pase mRNA expression was reported to be lower in liver biopsy samples from NAFLD patients [16]. Furthermore, to our knowledge, no studies have analyzed the associations among IRSs and these key molecules for gluconeogenesis and lipogenesis in human liver.

In the present study, we examined mRNA expressions of key molecules for insulin signaling, gluconeogenesis and lipogenesis in human liver biopsy samples and analyzed associations of these molecules with each other and with detailed pathological and clinical biochemistry data. Hepatic IRS-2 mRNA was significantly lower in SS and NASH patients than in controls, while hepatic PEPCK and G6Pase mRNA levels in the SS and NASH patients were significantly higher than those in the control group. Moreover, most importantly, hepatic IRS-2 mRNA levels were negatively associated with PEPCK and G6Pase mRNA. Thus, our integrated analyses of multiple gene expressions suggest that decreased IRS-2 plays an important role in the development of hepatic insulin resistance in humans with NAFLD. Protein expressions were not examined, which is a limitation of this study, due to the small amounts of liver biopsy material available. However, mRNA expression the levels of these key molecules, including IRS-1 [30], IRS-2 [30], PEPCK [31], G6Pase [32], GCK [33] and FAS [34], reportedly reflect their functions. In addition, insulin is well known to affect these key metabolic enzymes at mRNA expression levels. Therefore, analyses of mRNA expression levels may allow a conclusion to be drawn as to whether selective insulin resistance occurs in human NAFLD. Our human findings are consistent with those obtained from experimental animal models with hepatic steatosis and obesity. Therefore, the results obtained from experiments using animal models are likely to also hold true for the pathophysiology of human hepatic insulin resistance.

In addition, there were no differences in the expressions of IRS-2, PEPCK or G6Pase between the SS and the NASH groups in this study. The decrease in IRS-2 and the increases in PEPCK and G6Pase were associated with the histological degrees of steatosis and lobular inflammation, although IRS-2 was not associated with the degree of either fibrosis or ballooning. Moreover, serum liver enzymes were associated with hepatic expressions of IRS-2, PEPCK and G6Pase. In previous studies, the controls were morbidly obese subjects [14] and patients with elevated liver enzymes [16]. In contrast, our control group consisted of healthy living donors for liver transplantation, in whom normal biochemical liver markers and histological findings had been confirmed. Thus, our integrated analyses of human NAFLD cases, including pathological scoring, biochemical data and gene expression data, and detailed comparisons with healthy controls enabled us to show that alterations in IRS-2, PEPCK and G6Pase expressions are concurrently induced in the early stage of NAFLD, when histological steatosis and lobular inflammation, but not ballooning and fibrosis, manifest.

**Fig. 3:**
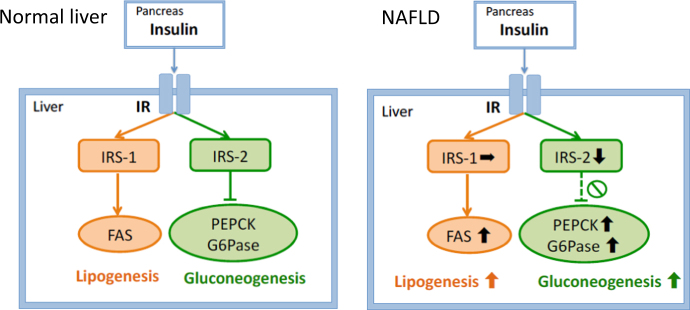
Insulin signaling in the liver of subjects with normal liver and NAFLD. The expression of insulin receptor substrates-2 (IRS-2) is decreased, while the expression of phosphoenolpyruvate carboxykinase (PEPCK) and glucose-6-phosphatase (G6Pase) are increased in patients with NAFLD. In contrast, the expression of IRS-1 is unaltered and the expression of fatty acid synthase (FAS) is increased in patients with NAFLD

Another important finding of the present study is that selective insulin resistance was demonstrated and differential contributions of IRS-1 and IRS-2 were suggested in human NAFLD subjects. FAS mRNA levels tended to be increased in the SS group and were significantly increased in the NASH group, indicating that insulin signaling for lipogenesis is activated in NAFLD despite impaired suppression of gluconeogenetic enzymes. Insulin reportedly increases SREBP-1c gene expression and activates SREBP-1c proteolytic cleavage in the liver [41]. After proteolytic cleavage, the mature N-terminal SREBP-1c translocates into the nucleus, where it binds to SRE of the FAS promoter. FAS is transcriptionally regulated by SREBP-1c in response to insulin [41]. Through this mechanism, insulin signals may up-regulate FAS in the liver. Indeed, FAS expression, as well as, that of SREBP-1c is reportedly increased in subjects with NAFLD [42]. The present study suggests that IRS-1, rather than IRS-2, is involved in the insulin-mediated FAS upregulation in hepatocytes. In contrast to IRS-2, no significant differences were observed in IRS-1 expressions among the control, SS and NASH groups. It should be noted that FAS expression correlated strongly with IRS-1, suggesting that IRS-1 signaling plays an important role in FAS expression. Furthermore, no histological scores were associated with either IRS-1 or FAS, suggesting that IRS-1 signaling is not impaired in patients with NAFLD. Taken together with the report indicating the role of IRS-1 as the inducer of GCK and SREBP-1c, shown in rat hepatocytes [43] and liver-specific IRS-1 knockout mice [44], persistent expression of IRS-1 and hyperinsulinemia in NAFLD may enhance FAS upregulation in the steatotic liver. These results indicate that insulin signaling for lipogenesis via IRS-1 is maintained despite impairment of the insulin signaling which favors suppression of gluconeogenesis via IRS-2 (Fig. 3). To the best of our knowledge, this is the first report showing selective insulin resistance in the human liver and further indicating that the differences in the expression patterns of IRS-1 and IRS-2 are likely to be involved in the underlying mechanism. During preparation of this manuscript, it was reported that hepatic IRS-2 knockout mice develop selective insulin resistance [9]. The study, employing the murine gene targeting technique, supports our findings in humans.

We have obtained novel findings shedding light on IR expression in the liver. In our study, hepatic IR expressions were higher in the SS and NASH groups than in the controls. IR mRNA has two isoforms due to alternative splicing variation of exon 11 of the IR gene. These IR isoforms can form either homodimers (IR-A/IR-A, IR-B/IR-B) or a heterodimer (IR-A/IR-B). The IR-A (exon 11-) isoform reportedly has a mitogenic property, while the IR-B (exon 11 + ) isoform is predominantly involved in glucose metabolism [45]. IR-B is reported to be the dominant (~80%) isoform in the human liver [46] but IR-A/IR-A [45] and the IR-A/IR-B [47] have higher affinities for IGF-II than IR-B/IR-B. In our study, similar results were obtained in healthy controls but the IR-B/IR-A ratio was significantly decreased in the SS and NASH groups. The increase in the proportion of IR-A may stimulate mitogenic signaling, which might contribute to carcinoma development, one of the major complications of NAFLD, although further studies are needed to elucidate the actions of these IR isoforms in NAFLD patients. In addition, a decrease in the proportion of IR-B may also contribute to the development of hepatic insulin resistance, which would in turn impact glucose metabolism.

In the present study, we examined hepatic gene expressions of key molecules involved in insulin signaling, lipogenesis and gluconeogenesis in both healthy subjects and patients with NAFLD. Our integrated analyses encompassed both these findings and detailed pathological and clinical biochemistry data. These analyses revealed that selective insulin resistance occurs in the human liver, and that differential expression patterns of IRS-1 and IRS-2 may contribute to the underlying mechanism. In addition, the IR-A isoform was up-regulated in NAFLD patients. Collectively, these results are significant for understanding the pathophysiology of NAFLD and its associated metabolic disorders.

## Electronic supplementary material

Supplemental Figure 1

Supplemental Figure 2
