# Supplementary material for: Selective insulin resistance with differential expressions of IRS-1 and IRS-2 in human NAFLD livers
Source: Int J Obes (Lond). 2018 May 1;42(9):1544–55. doi: 10.1038/s41366-018-0062-9 (PMC6160396; doi:10.1038/s41366-018-0062-9)
Supplement: Supplementary file 1 — Supplemental Figure 1 [file 41366_2018_62_MOESM1_ESM.pptx]

## Slide 1
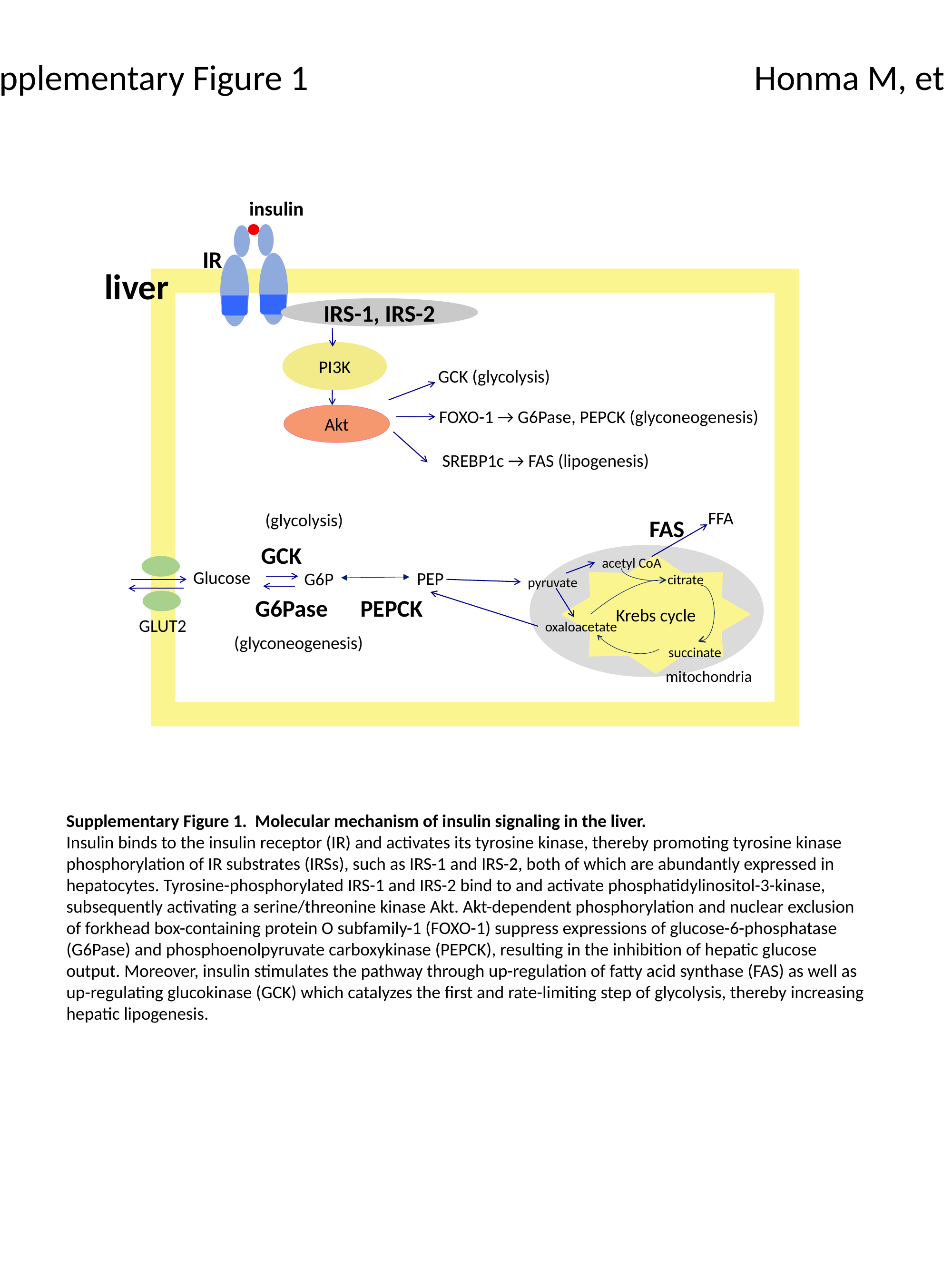

Supplementary Figure 1　　　　　　　　　　　　Honma M, et al.
insulin
IR
liver
IRS-1, IRS-2
PI3K
GCK (glycolysis)
FOXO-1 → G6Pase, PEPCK (glyconeogenesis)
Akt
SREBP1c → FAS (lipogenesis)
FFA
(glycolysis)
FAS
GCK
ぴ
acetyl CoA
Krebs cycle
Glucose
PEP
G6P
>
citrate
pyruvate
PEPCK
G6Pase
>
GLUT2
oxaloacetate
>
(glyconeogenesis)
succinate
mitochondria
Supplementary Figure 1. Molecular mechanism of insulin signaling in the liver.
Insulin binds to the insulin receptor (IR) and activates its tyrosine kinase, thereby promoting tyrosine kinase phosphorylation of IR substrates (IRSs), such as IRS-1 and IRS-2, both of which are abundantly expressed in hepatocytes. Tyrosine-phosphorylated IRS-1 and IRS-2 bind to and activate phosphatidylinositol-3-kinase, subsequently activating a serine/threonine kinase Akt. Akt-dependent phosphorylation and nuclear exclusion of forkhead box-containing protein O subfamily-1 (FOXO-1) suppress expressions of glucose-6-phosphatase (G6Pase) and phosphoenolpyruvate carboxykinase (PEPCK), resulting in the inhibition of hepatic glucose output. Moreover, insulin stimulates the pathway through up-regulation of fatty acid synthase (FAS) as well as up-regulating glucokinase (GCK) which catalyzes the first and rate-limiting step of glycolysis, thereby increasing hepatic lipogenesis.
