# Supplementary material for: Selective insulin resistance with differential expressions of IRS-1 and IRS-2 in human NAFLD livers
Source: Int J Obes (Lond). 2018 May 1;42(9):1544–55. doi: 10.1038/s41366-018-0062-9 (PMC6160396; doi:10.1038/s41366-018-0062-9)
Supplement: Supplementary file 2 — Supplemental Figure 2 [file 41366_2018_62_MOESM2_ESM.pptx]

## Slide 1
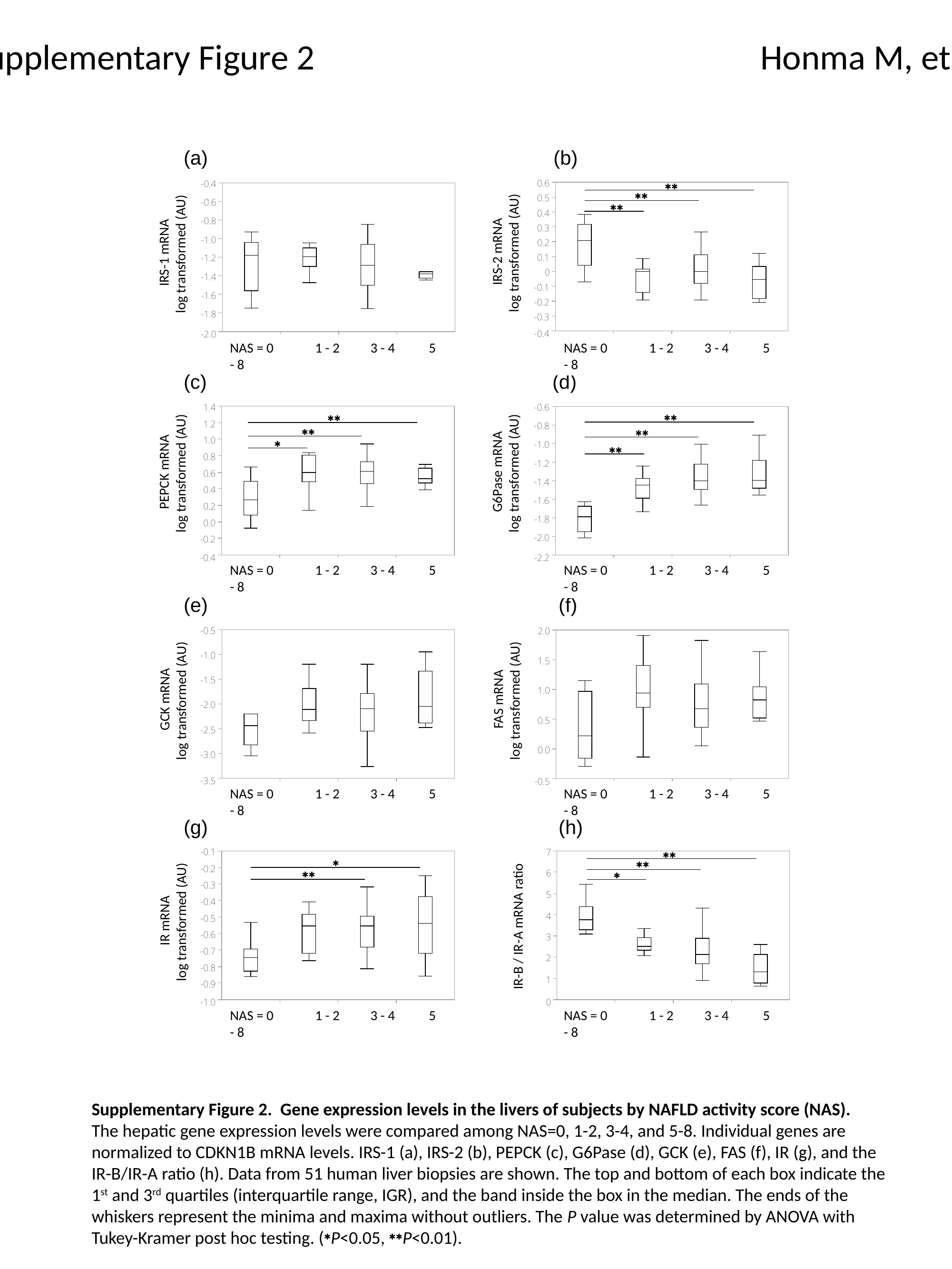

Supplementary Figure 2　　　　　　　　　　　　Honma M, et al.
(a) 　　　　 (b)
✱✱
✱✱
✱✱
IRS-2 mRNA
log transformed (AU)
IRS-1 mRNA
log transformed (AU)
NAS = 0　　 1 - 2 3 - 4 5 - 8
NAS = 0　　 1 - 2 3 - 4 5 - 8
(c) 　　　　 (d)
✱✱
✱✱
✱✱
✱✱
✱
✱✱
G6Pase mRNA
log transformed (AU)
PEPCK mRNA
log transformed (AU)
NAS = 0　　 1 - 2 3 - 4 5 - 8
NAS = 0　　 1 - 2 3 - 4 5 - 8
(e) 　　　　 (f)
FAS mRNA
log transformed (AU)
GCK mRNA
log transformed (AU)
NAS = 0　　 1 - 2 3 - 4 5 - 8
NAS = 0　　 1 - 2 3 - 4 5 - 8
(g) 　　　　 (h)
✱✱
✱
✱✱
✱✱
✱
IR mRNA
log transformed (AU)
IR-B / IR-A mRNA ratio
NAS = 0　　 1 - 2 3 - 4 5 - 8
NAS = 0　　 1 - 2 3 - 4 5 - 8
Supplementary Figure 2. Gene expression levels in the livers of subjects by NAFLD activity score (NAS).
The hepatic gene expression levels were compared among NAS=0, 1-2, 3-4, and 5-8. Individual genes are normalized to CDKN1B mRNA levels. IRS-1 (a), IRS-2 (b), PEPCK (c), G6Pase (d), GCK (e), FAS (f), IR (g), and the IR-B/IR-A ratio (h). Data from 51 human liver biopsies are shown. The top and bottom of each box indicate the 1st and 3rd quartiles (interquartile range, IGR), and the band inside the box in the median. The ends of the whiskers represent the minima and maxima without outliers. The P value was determined by ANOVA with Tukey-Kramer post hoc testing. (✱P<0.05, ✱✱P<0.01).
